# Identification of the role of C/EBP in neurite regeneration following microarray analysis of a *L. stagnalis *CNS injury model

**DOI:** 10.1186/1471-2202-13-2

**Published:** 2012-01-04

**Authors:** Mila Aleksic, Zhong-Ping Feng

**Affiliations:** 1 Department of Physiology, Faculty of Medicine, University of Toronto, 1 King's College Circle, Toronto, Ontario, M5S 1A8, Canada

## Abstract

**Background:**

Neuronal regeneration in the adult mammalian central nervous system (CNS) is severely compromised due to the presence of extrinsic inhibitory signals and a reduced intrinsic regenerative capacity. In contrast, the CNS of adult *Lymnaea stagnalis (L. stagnalis)*, a freshwater pond snail, is capable of spontaneous regeneration following neuronal injury. Thus, *L. stagnalis *has served as an animal model to study the cellular mechanisms underlying neuronal regeneration. However, the usage of this model has been limited due to insufficient molecular tools. We have recently conducted a partial neuronal transcriptome sequencing project and reported over 10,000 EST sequences which allowed us to develop and perform a large-scale high throughput microarray analysis.

**Results:**

To identify genes that are involved in the robust regenerative capacity observed in *L. stagnalis*, we designed the first gene chip covering ~15, 000 *L. stagnalis *CNS EST sequences. We conducted microarray analysis to compare the gene expression profiles of sham-operated (control) and crush-operated (regenerative model) central ganglia of adult *L. stagnalis*. The expression levels of 348 genes were found to be significantly altered (p < 0.05) following nerve injury. From this pool, 67 sequences showed a greater than 2-fold change: 42 of which were up-regulated and 25 down-regulated. Our qPCR analysis confirmed that CCAAT enhancer binding protein (C/EBP) was up-regulated following nerve injury in a time-dependent manner. In order to test the role of C/EBP in regeneration, C/EBP siRNA was applied following axotomy of cultured *Lymnaea *PeA neurons. Knockdown of C/EBP following axotomy prevented extension of the distal, proximal and intact neurites. *In vivo *knockdown of C/EBP postponed recovery of locomotory activity following nerve crush. Taken together, our data suggest both somatic and local effects of C/EBP are involved in neuronal regeneration.

**Conclusions:**

This is the first high-throughput microarray study in *L. stagnalis*, a model of axonal regeneration following CNS injury. We reported that 348 genes were regulated following central nerve injury in adult *L. stagnalis *and provided the first evidence for the involvement of local C/EBP in neuronal regeneration. Our study demonstrates the usefulness of the large-scale gene profiling approach in this invertebrate model to study the molecular mechanisms underlying the intrinsic regenerative capacity of adult CNS neurons.

## Background

Injuries of the central nervous system (CNS) can lead to devastating and irreversible loss of function because adult mammalian CNS neurons have a limited regenerative capacity [1-5]. This is partially due to an age dependent reduction in the intrinsic regenerative potential of CNS neurons [6-9]. In contrast, some adult invertebrate neurons are capable of spontaneous regeneration following injury [10,11]. *Lymnaea stagnalis *(*L. stagnalis*) has served as a critical model system to study nerve regeneration because of its ability to spontaneously regenerate and restore function in the adult [12-16]. Specifically, identified adult *Lymnaea *neurons can be isolated individually and maintained in culture, allowing for axonal outgrowth and functional synapse formation between appropriate synaptic partners [17-19]. *L. stagnalis *neural genes include homologues of well known transcription factors, genes involved in neurotransmission, axon guidance and signalling pathways [20]. Taking advantage of this model system, a number of groups demonstrated the roles of neurotrophins [14,21], and neurotransmitters [12,22] on neurite outgrowth and regeneration. Moreover, retinoic acid (RA) isomers have been identified in adult *Lymnaea *neurons, and 9-*cis*-RA has a novel role in chemoattraction and growth cone guidance [23-25]. Since many molecular mechanisms are conserved across species, the identification of molecules involved in neuronal regeneration in *L. stagnalis *will aid our understanding of factors which may promote regeneration in the mammalian CNS.

One main limitation of using *L. stagnalis *in genetic functional studies of neuronal regeneration has been a lack of large-scale screening tools, such as microarray analysis [26,27]. To circumvent this limitation, we have recently sequenced more than 10, 000 ESTs (expression sequence tags) from the CNS transcriptome of *L. stagnalis *[20] and established the largest neuronal EST database in *Lymnaea *(http://www.lymnaea.org). In combination with the availability of the microarray technology [28,29], these gene sequences provide us with the opportunity to perform a high-throughput screening for altered gene expression levels following nerve injury in *L. stagnalis*. In this study, we have designed the first microarray chip covering 10, 333 known *L. stagnalis *genes to profile the gene expression patterns following CNS injury. We identified 348 genes that were differentially regulated following CNS crush. Using real-time PCR (qPCR) analysis we confirmed that the gene expression level of CCAAT enhancer binding protein (C/EBP), a transcription factor, is up-regulated following nerve injury. Knockdown of C/EBP following axotomy of cultured PeA cells lead to retraction of the distal, proximal and intact neurites. Our data suggest that C/EBP plays a crucial role in neuronal regeneration of *Lymnaea *neurons via both somatic and local mechanisms.

## Results

### CNS injury model of *L. stagnalis*

**Figure 1:**
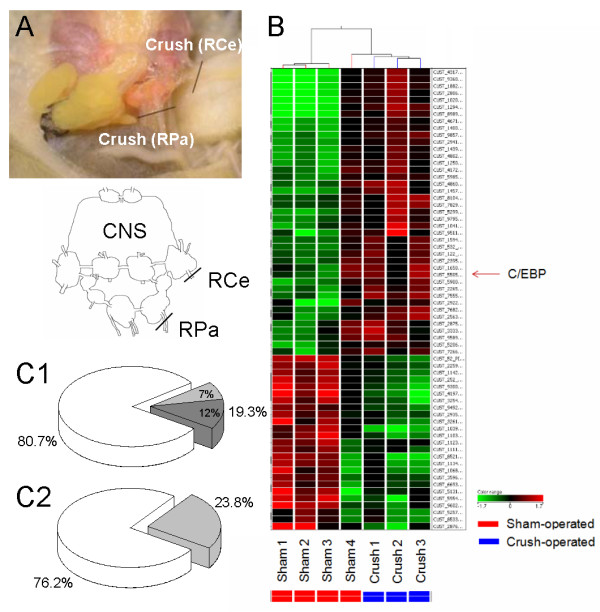
**Experimental model and changes in gene expression following injury**. (**A**) The dorsal surface of *L. stagnalis *CNS and CNS injury protocol: both right parietal (RPa) nerves and right cerebral nerves (RCe) were crushed with fine forceps. Inset: a schematic diagram of the ganglia and location of injury (black lines). (**B**) Heat map representing the hierarchical clustering of 67 genes showing significant differential expression (P < 0.05). The expression pattern of each gene in the sham and crashed groups is displayed as a horizontal strip. For each gene, the expression ratio of the crush-operated to the sham-operated experiments is represented by the green and red scale at the bottom of the figure. The genes are numbered on the right, and the experimental groups are labelled on the bottom. (**C**) Ratio of the regulated EST sequences 3 hrs following CNS injury in *L. stagnalis*. (C1) The signal intensity of 348 genes or ESTs was differentially regulated. Open pie (80.7%) represents genes/EST's changes < 2.0-fold in signal intensity but significant (P < 0.05); grey pie (19.3%): changes ≥ 2.0-fold (P < 0.05). Dark grey: up-regulated genes (12%), and light grey: down regulated genes (7%). (C2) Ratio of the 67 differentially regulated genes, 16 (23.8%) genes have orthologous with known functions related to development, survival or signal transduction, whereas 51 (76.2%) genes have no orthologue.

In order to profile the gene expression pattern following injury of the central nervous system, we modified an established [12,14] nerve injury model by crushing the right parietal (RPa) and right cerebral nerves (RCe) (Figure 1A). In control groups, sham operations were performed without nerve injury.

### Identification of differentially expressed genes

To search for genes that may be involved in neuronal regeneration or degeneration, microarray analyses were carried out to compare the gene expression levels between the sham-operated and crush-operated CNS models (Figure 1A, inset). We used an unpaired t-test for comparison of the 15,000 array signals between two groups: sham- and crush-operated groups. This analysis resulted in the selection of 348 genes or ESTs (See Additional File 1) that were differentially regulated 3 hours following CNS injury (p < 0.05). Further screening for genes with a fold-change > 2 resulted in a final group of 67 differentially expressed genes. Figure 1B shows a heat map representing the hierarchical clustering of the 67 genes with significant differential expression levels between the control and injured groups. Overall, more genes were up-regulated (n = 42) than that of which were down-regulated (n = 25), suggesting that diverse regulatory pathways and biological processes are activated within the first 3 hours following injury (Figure 1C1). Among the 67 regulated genes, 16 have been identified with known functions related to development, survival or signal transduction; the functions of the majority of genes (51) have not yet been described (Figure 1C2).

### Gene expression levels of C/EBP change in a time-dependent manner following injury

**Figure 2:**
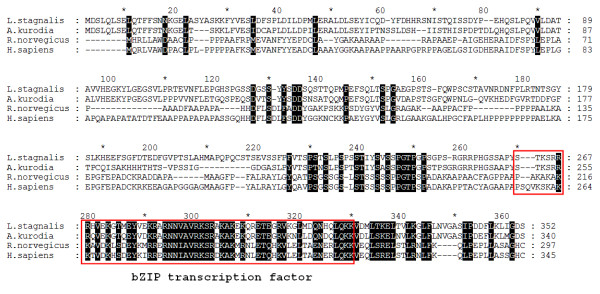
**Protein sequence alignment of C/EBP**. Amino acid alignment between CCAAT enhancer binding protein from *L. stagnalis *[GenBank accession#: BAD16556], *A. kurodia *[GenBank#: AAG61258], *R. norvegicus *[GenBank#: AAI29072] and *H. sapian *[GenBank#: EAW75629] C/EBP. The high degree of sequence similarity at the DNA-binding domain indicates that C/EBP has a conserved functional domain across species.

**Figure 3:**
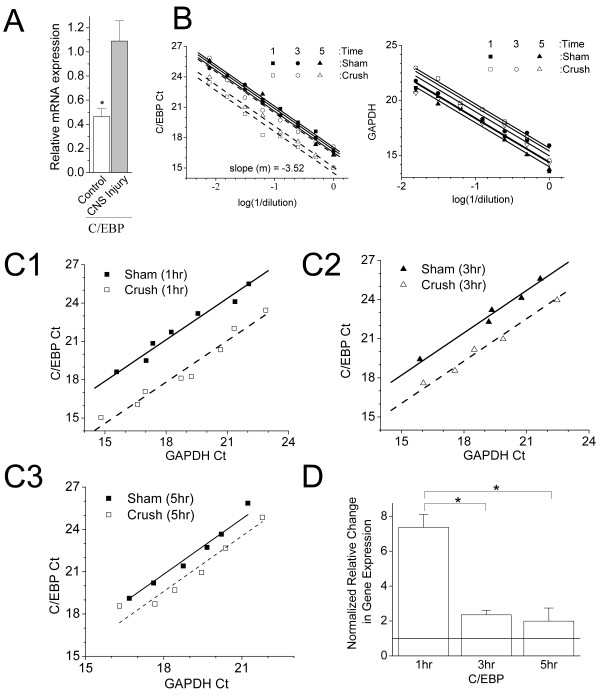
**Time-dependent changes in C/EBP mRNA expression following nerve injury**. Real-time PCR was performed with specific primers for C/EBP between sham-operated and crush-operated *L. stagnalis *CNS at 1 hr, 3 hrs, and 5 hrs post injury. (A) Relative mRNA levels of C/EBP vs GADPH increased significantly following CNS injury as compared to the sham-operated controls at 3 hrs (2.35 ± 0.26, n = 5) (t = -3.5, df = 8, pP < 0.05). (B) Standard curves of C/EBP and GAPDH from 6 independent samples including sham-operated and crush-operated *L. stagnalis *CNS at 1 hr, 3 hrs, and 5 hrs post injury. The PCR efficiency of the primers was estimated by the slope (m) = -1/log(efficiency). The expression GAPDH was unchanged following CNS injury, and therefore CAPDH was used as an internal control. (C) Representative Ct-Ct correlation plots between C/EBP and the control gene, GADPH, at three different time points following injury. C1: 1 hr; C2: 3 hrs; C3: 5 hrs. Relative expression ratio between C/EBP and GADPH was estimated as Y_intercept _= -log(ratio)/log(efficiency C/EBP). (D) Relative gene expression of C/EBP vs. GADPH normalized to corresponding control samples. C/EBP increased in a time-dependent manner. 1 hr: 7.36 ± 0.73 (n = 5); 3 hrs: 2.35 ± 0.26 (n = 5), and 5 hrs: 1.98 ± 0.75 (n = 5). * indicates significant differences: ANOVA: F_(2,12) _= 17.4, p < 0.05. All data are presented as mean ± S.E.M.

The microarray analysis indicated that the mRNA level of CCAAT enhancer binding protein, C/EBP, was significantly increased 3 hours following CNS injury, as compared to the sham control group. A protein sequence alignment of C/EBP orthologues from *L. stagnalis, Aplysia*, rat, and human revealed that C/EBP has a high homology in the DNA-binding domain, suggesting that C/EBP may have similar biological functions in these species (Figure 2). To confirm that C/EBP is indeed up-regulated following nerve injury at 3 hours, we performed conventional RT-PCR analysis. We found that the expression level of C/EBP mRNA significantly increased, compared to the control gene GAPDH, in the injured CNS group (2.35 ± 0.26 fold; n = 5, p < 0.05) over the sham groups (Figure 3A).

To study its temporal expression pattern, we further measured the gene level of C/EBP at 1 hour, 3 hours and 5 hours post CNS injury using qPCR analysis. The standard curves for C/EBP are shown in Figure 3B with a slope of -3.52, indicating the PCR efficiency for the primers are sufficient (Figure 3B). To visualize the gene expression level between the injured and sham groups, the threshold cycles (C_t_) of C/EBP (the target gene) and GAPDH (the control gene) were plotted against each other, as described previously [30]. Figure 3C shows the representatives of C_t_-C_t _correlation plots between the C/EBP and GADPH gene pair at the three different time points (1 hr, 3 hrs, 5 hrs). The slopes of the plots were consistent in all experimental samples, indicating the equality in amplification efficiencies between the primers of the gene pair. The Y-intercepts (- log(R)/log(E_g_)) of these plots were smaller in the injury group (crush) than in the controls (sham) at all the times tested, indicating the relative expression level of C/EBP mRNA increased following injury. Specifically, C/EBP mRNA was initially increased by 7.36 ± 0.73, (n = 5) at 1 hr after CNS injury, but appeared to reduce to 2.35 ± 0.26 (n = 5) by 3 hrs and 1.98 ± 0.75, (n = 5) by 5 hrs, albeit at levels that are still significantly greater than in sham-operated (control) animal (Figure 3D). These results suggest that elevation of C/EBP mRNA expression may be necessary for transcriptional induction of regeneration associated genes, and thus likely to be a direct target of injury induced signalling cascades that promote neuronal repair.

### The role of C/EBP in axonal elongation following axotomy

**Figure 4:**
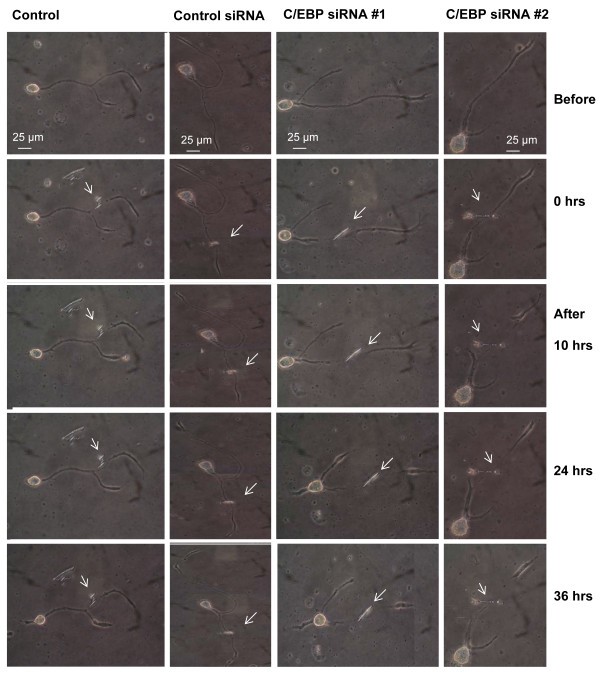
**Knockdown of C/EBP reduced net growth of the distal, proximal, and intact neurites following axotomy**. Representatives of transected axons in culture. Immediately following axotomy (t = 0), either CM, control siRNA, C/EBP siRNA #1, or C/EBP siRNA #2 was added into culture medium at a final concentration of 7 nM, immediately after neurite transection. The neurons were observed over additional 36 hours and images were taken at 10, 24 and 36 hours as indicated. Arrows denote the transection site.

**Figure 5:**
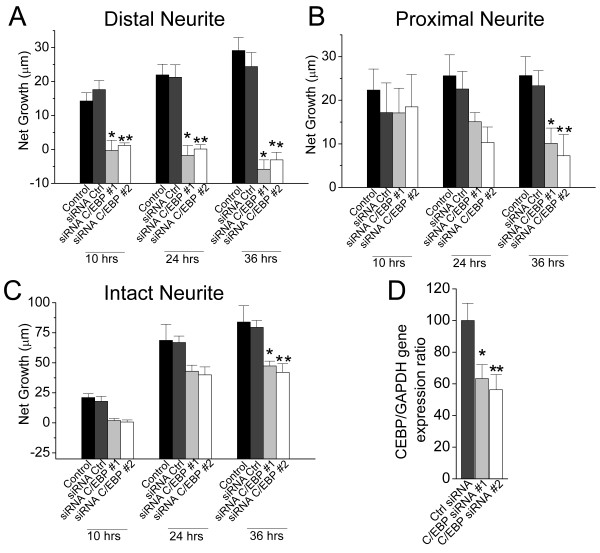
**Differential effects of C/EBP knockdown on the distal, proximal, and intact neurites following axotomy**. The length of the distal, proximal, or intact neurite of injured cells was measured at three time points (10 hrs, 24 hrs, 36 hrs) after control or C/EBP siRNA treatment, which was given immediately after axotomy (t = 0). (A) The distal neurite. The net changes in the C/EBP siRNA #1 or #2 treated cells were significantly reduced as compared to that in the control CM or control siRNA treated cells (n = 14; ANOVA: at 10 hrs, F_(3,52) _= 14.4, p < 0.05; at 24 hrs, F_(3,52) _= 20.1, P < 0.05; at 36 hrs, F_(3,52) _= 29.7, p < 0.05). (B) The proximal neurite. A significant reduction in outgrowth was observed only at 36 hrs after axotomy in the C/EBP siRNA #1 or #2 treated cells as compared to control CM and control siRNA treated cells (n = 14; ANOVA: F_(3,52) _= 5.1, p < 0.05). (C) The intact neurite. A significant reduction in outgrowth was only observed at 36 hrs in the C/EBP siRNA #1 or #2 treated cells as compared to control CM and control siRNA treated cells (n = 11; ANOVA: F_(3,40) _= 6.5, p < 0.05). (D) Relative gene expression level of C/EBP vs GAPDH was significantly reduced in the C/EBP siRNA #1 (63.34 ± 8.8%) and #2 (56.25 ± 9.4%) (n = 5, ANOVA: F_(2,12) _= 5.7, p < 0.05) groups as compared to control siRNA group. All data are presented as mean ± S.E.M.. * indicates p < 0.05.

The increased expression of C/EBP mRNA following nerve injury *in vivo *suggests that this protein may be an upstream gene regulator of either pro-regenerative genes or responsive genes for cell injury. To elucidate the functional significance of C/EBP mRNA following nerve injury, we examined the effect of C/EBP on axonal outgrowth and regeneration of PeA neurons in culture using a siRNA gene silencing approach as previously described [31-33]. Axotomy was performed on cultured PeA neurons after 24 hrs which had undergone adequate neurite outgrowth (Figure 4). Immediately following axotomy, cells were treated with either conditioned medium (CM), control siRNA, C/EBP siRNA#1 or C/EBP siRNA#2. The lengths of the proximal and distal ends of the axotomized neurite, as well as an intact neurite were measured at various times over a period of 36 hrs (Figure 4). The net changes of the neurites following treatments were compared, as previously described [33]. Figure 5A shows that the distal end axons did not show additional growth following treatments with either C/EBP siRNA#1 (10 hrs: -0.3 ± 3.0 μm; 24 hrs: -1.8 ± 2.9 μm; 36 hrs: -5.9 ± 2.8 μm. n = 14), or C/EBP siRNA#2 (10 hrs: 1.2 ± 0.7 μm; 24 hrs: 0.1 ± 1.3 μm; 36 hrs: -3 ± 2.2 μm. n = 14) treated cells, whereas that continuously elongated in control CM (10 hrs: 14.2 ± 2.4 μm; 24 hrs: 21.9 ± 3.1 μm; 36 hrs: 29.1 ± 3.9 μm. n = 14) or control siRNA (10 hrs: 17.6 ± 2.7 μm; 24 hrs: 21.2 ± 3.7 μm; 36 hrs: 24.4 ± 4 μm. n = 14) treated cells following axotomy. The net changes in the distal axons were significant between the C/EBP siRNAs and the control CM or control siRNA groups (p < 0.05, for all three time points). In contrast, the net increases in length of the proximal neurites over 36 hrs are 25.6 ± 4.3 μm (n = 14) in control CM, and 23.3 ± 4.3 μm (n = 14) in control siRNA treated cells, whereas that was significantly reduced in C/EBP siRNA #1 (10.12 ± 3.5 μm, n = 14) or C/EBP siRNA #2 (7.2 ± 4.8 μm, n = 14) treated cells, although no significant difference was found at the early hours (10 or 24 hrs) (Figure 5B). Similarly, the net growth of intact neurites was only significantly reduced in C/EBP siRNA #1 (47.4 ± 4 μm, n = 11) and C/EBP siRNA #2 (42 ± 7.1 μm, n = 11) treated cells as compared to control CM (83.9 ± 13.5 μm, n = 11) and control siRNA treated cells (79.5 ± 5.7 μm, n = 11) 36 hrs after treatment (Figure 5C). These findings suggest that neuronal C/EBP is involved in neurite outgrowth. Sufficient C/EBP levels are required for maintaining the integrity of the distal axon. Due to the application of siRNAs following axotomy, the differences found in the distal axons may be associated with local protein synthesis [33,34].

### C/EBP siRNA treatment hinders locomotion recovery *in vivo *following CNS injury

**Figure 6:**
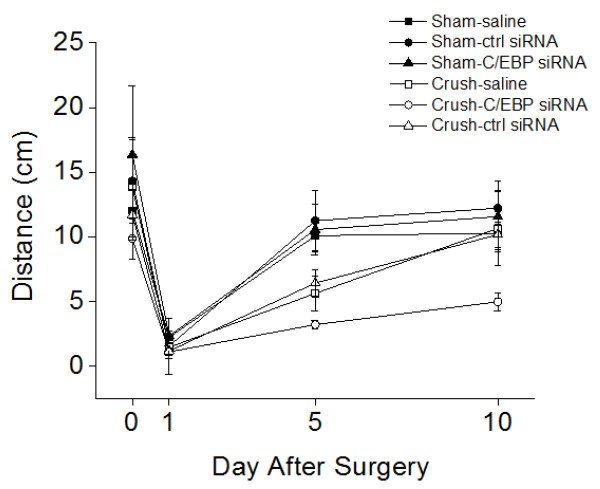
**C/EBP siRNA treatment hinders recovery of locomotion activity of the snails following CNS injury**. Sham-operated or crush-operated snails were injected with either saline, control siRNA or C/EBP siRNA. The distance that the injured snails crawled in 10 min was measured at various time points (1 day, 5 days, and 10 days) following the nerve crush procedure. On the 10^th ^day, the crush-operated C/EBP siRNA group crawled an average of 4.99 ± 0.71 cm (n = 5) per 10 min which was reduced as compared to the sham-operated saline (10.28 ± 1.28 cm, n = 6), control siRNA (12.23 ± 1.36 cm, n = 6), or C/EBP siRNA (11.56 ± 2.75 cm, n = 5), and crush-operated saline (10.65 ± 2.84 cm, n = 4) or control siRNA treated snails (10.17 ± 1.0 cm, n = 5). Data are presented as mean ± S.E.M. * indicates p < 0.05.

In order to determine whether C/EBP siRNA treatment has a role in an *in vivo *model, we compared the locomotory activity of sham-operated and crush-operated snails treated with saline, control siRNA and C/EBP siRNA#1. The distance that the snail crawled in 10 min was measured before and after nerve crush (1, 5 and 10 days post-crush), between all six groups. As shown in Figure 6, all groups had severely movement deficit following the nerve crush operation, but slowly gained their ability to crawl over the course of 10 days. However, after 10 days, the distance (per 10 min) measured from the crush-operated C/EBP siRNA#1 group (4.99 ± 0.71 cm, n = 3) was reduced as compared to that from the sham-operated saline (10.28 ± 1.28 cm, n = 6), control siRNA (12.23 ± 1.36 cm, n = 6) or C/EBP siRNA#1 (11.56 ± 2.75 cm, n = 5), as well as to that from the crush-operated saline (10.65 ± 2.84 cm, n = 4), or control siRNA treated snails (10.17 ± 1.0 cm, n = 5). These data suggest that C/EBP siRNA treatment disrupted the recovery of snails following nerve injury and support the notion that C/EBP knockdown impedes CNS regeneration *in vivo*, potentially through its effects on neurite regeneration.

## Discussion

In this study, we designed the first *Lymnaea *gene chip for microarray analysis and profiled the gene expression patterns during the early stages of the CNS repair following injury. We found that 67 genes were significantly regulated during the first three hours post injury, including the conserved gene transcription factor C/EBP. We further described the temporal gene regulation pattern of C/EBP following CNS injury using qPCR analysis. Reduction of C/EBP mRNA level by C/EBP siRNAs following axotomy suppressed outgrowth of the distal and somatic neurite *in vitro *at different time points, and disrupted the recovery of locomotory function of the snails following nerve injury *in vivo*. Our data further support the notion that C/EBP is required for nerve regeneration.

*L. stagnalis *has been used as a simple and reliable model to investigate genes involved in regeneration, due to its ability to functionally recover following CNS injury *in vivo*. For example, central ganglia cell (CGC) connections to buccal ganglia were crushed and as a result the feeding behaviour in animals was disrupted. Two weeks following the procedure the CGC regenerated, functional synapses formed between proper synaptic partners and the feeding behaviour was again observed [12]. *In vivo *CNS regeneration was also observed following axotomy of innervating axons to the pneumostome and surrounding areas which prevented the occurrence of lung respiration in 69% of snails. Several weeks following surgery, axonal regeneration leading to the reformation of functional synapses was observed and pneumostome opening returned to normal [13]. In this study, we used the modified CNS injury model in which both the right parietal (RPa) and the right cerebral nerves (RCe) were crushed (Figure 1A). We are interested in the gene regulation occurring at the early stages of CNS trauma, thus our *in vivo *experiments were completed within 5 hours following injury.

The ability to profile the expression of thousands of genes simultaneously makes microarrays an invaluable biomedical research tool [35]. A previous study used microarray approach to assess the genes involved in anti-inflammatory responses in a spinal cord injury model [28]. The anti-inflammatory response is an important component of secondary tissue damage following spinal cord injury. The microarray study allowed for identification of a conserved novel regulator of regeneration, and provided a new mechanism by which to initiate the regenerative response [29]. However, shortcomings of microarray analysis are associated with poor reliability [35-38] and specificity [35]. Due to the inherent sources of error in microarray technology, it is necessary to confirm the findings using an independent methodology from separate samples. Real-time quantitative PCR is a readily used technique to confirm microarray data [39-41]. In our study, we conducted qPCR analysis using different samples and verified that the expression of C/EBP was indeed up-regulated following nerve injury.

Recovery from nerve injury and neuronal regeneration are time-dependent events, from the initial inflammatory response to the development of the glial scar [42]. Inflammation, neurotransmitter dysfunction, ionic imbalance, cytoskeletal alteration and increased transcription occur as early as 3 hrs following spinal cord injury [41,43,44]. Nerve outgrowth and regeneration can be enhanced by steroids during a critical window within the first 6 hrs after nerve injury [45]. In the *C. elegans*, axons regenerate following axotomy *in vivo *and by 24 hrs fully functional synapses are formed [46]. We have recently demonstrated that *L. stagnalis *regeneration post injury *in vitro *can be seen as early as 3 hrs, and reformation of connections between the proximal and distal axon is observed by 24 hrs [47]. Deducing the gene regulation during the early response following neuronal injury is important in uncovering the molecular basis of neuronal regeneration. In this study, our microarray gene expression analysis identified 67 genes that were significantly regulated following CNS injury in *L. stagnalis*. We examined C/EBP gene expression at three different time points (1 hr, 3 hrs, 5 hrs) in order to understand how it is regulated temporally in the early stages of recovery and repair. Our results indicated that the C/EBP gene was up-regulated in a time-dependent manner following CNS injury (Figure 3). We further showed the locomotory activity of the control snails was severely disrupted immediately following nerve injury but recovered gradually over 10 days. C/EBP siRNA application prior to the nerve crush procedure disrupted the recovery of locomotory function of the snails (Figure 6), suggesting that C/EBP is required for nerve regeneration *in vivo*. Although our *in vitro *data suggest that sufficient C/EBP levels in neurons are required for axonal outgrowth, it is possible that an increased C/EBP expression in non-neuronal (phagocytic) cells involved in nerve regeneration and inflammatory responses also contributes to the observed recovery of locomotory activity in the injured snails.

C/EBPs belong to the basic leucine zipper DNA binding protein family and are transcription factors with diverse cellular roles, including regulating cell growth, differentiation, learning and memory, and apoptosis [48-50]. C/EBPs are also responsive to brain injury and ischemia [51,52]. Six isoforms of C/EBP have been found in mammals [53]. C/EBPβ is highly homologous to *Lymnaea *C/EBP (*L*C/EBP) [54]. C/EBPβ was first identified as a mediator of the inflammatory response and IL-6 signalling through its binding to IL-6 response elements in the promoters of acute phase response genes TNF, IL-8, and G-CSF [55]. C/EBP expression is enriched in neurons. It is up-regulated following brain injury in various neuronal regenerative animal models [56-58]. Its expression is regulated by transcription factors such as CREB and NFIL-3 [59-61]. *Aplysia *nerve injury activates axoplasmic kinase, RISK-1, which phosphorylates apC/EBPβ and initiates the binding of apC/EBPβ to the ERE enhancer site *in vitro*. Increases in RISK-1 and apC/EBP were detected in injured neurons [59]. In leech, C/EBP mRNA was increased during neuronal regeneration [60]. In this study, we show that C/EBP mRNA expression is increased following CNS injury in a time-dependent manner. The 7-fold increase in C/EBP mRNA at 1 hr following injury is likely related to the inflammatory response. The sustained 2 fold increase over the 5 hr period suggests that C/EBP may be essential for transcriptional induction of regeneration associated genes. Knockdown of C/EBP mRNA expression following axotomy by target-gene siRNA caused a reduction in neurite outgrowth in both distal and proximal axons at different time points. Due to that siRNAs were applied following axotomy, our data of C/EBP siRNAs causing suppression of distal neurites suggests that local C/EBP mRNA is likely involved in neuronal regeneration/elongation. In contrast to the distal section of neurites which may rely on local protein synthesis to grow, the cell somata may continuously provide complimentary mechanisms to sustain C/EBP levels and maintain neurite properties. The delayed effect of C/EBP siRNAs on the proximal regrowth (Figure 5B) further supports the notion that C/EBP is required for axonal regeneration.

**Figure 7:**
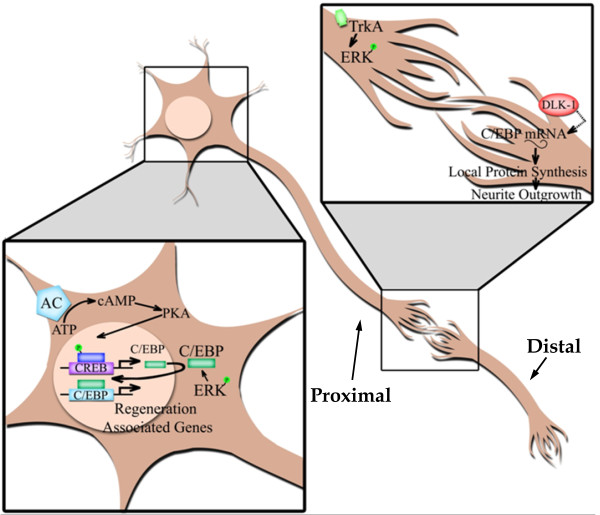
**Schematic illustration of the potential mechanisms of C/EBP action in maintaining distal axon integrity following axotomy**. C/EBP mRNA is expressed in both the soma and axon [34,56]. In the soma, C/EBP functions as a transcription factor and results in the transcription of regenerative-associated genes, such as α-tubulin and GAP-43, via a cAMP/PKA/CREB dependent signalling pathway [56]. Axon injury also activates an ERK-dependent pathway that results in the phosphorylation of C/EBP and further activation of pro-regenerative genes [59]. In both proximal and distal axons, C/EBP mRNA is stabilized by DLK-1 following axotomy [34]. Local protein synthesis of C/EBP in the distal axon may be required for regeneration or to prevent degeneration of distal axons following injury.

CEBP-1, a member of the C/EBP family in the *C. elegans*, is a direct effecter of the DLK-1 cascade [34]. CEBP-1 mRNA has been found in axons and presynaptic regions, and is stabilized via its 3'UTR by activation of the dual-leucine zipper Kinase-1 (DLK-1) pathway [34]. The role of C/EBP in axon regeneration is suggested via dual-leucine zipper kinase MAPKKK (DLK-1)-dependent pathway [34]. Neuronal regeneration associated proteins such as α-tubulin and GAP-43, are transcriptional targets of C/EBP [48,56,62]. Following nerve injury, up-regulation of C/EBPβ mRNA and C/EBP phosphoprotein coincides with increases in the transcription of the α-tubulin promoter in the wild-type but not in C/EBPβ^-/- ^mice [56]. DLK-1 acts in a MAPK cascade with MAPMKKK-4 and p38 kinase [63]. Both the DLK-1 pathway and CEBP-1 were necessary for regenerative growth of adult touch neurons [34]. We thus proposed a model by which the DLK-1 pathway regulates C/EBP mRNA stability in both distal and proximal axons. Both somatic and local C/EBP levels are required for the regenerative properties of snail neurons (Figure 7). Local protein synthesis has been shown to increase in injured axons and thus play an important role in the regeneration-degradation process [64-66]. Our study provided the first evidence that not only the somatic but also local regulation of C/EBP mRNA in axons is important for outgrowth and regeneration in *L. stagnalis*.

## Conclusions

This is the first microarray study in *L. stagnalis*, allowing us to identify genes that are differentially regulated following CNS injury. These genes may be involved in regeneration and provide a new database for the study of specific proteins and genes in response to CNS injury. We also identified the role of the transcription factor C/EBP in neuronal regeneration following axotomy of adult neurons *in vitro*, and provided new insights into the potential targets for CNS injury.

## Methods

### Animals

Fresh water pond snails, *L. stagnalis*, were obtained from culture at the Free University, Amsterdam, and maintained in the laboratory under standard conditions at the University of Toronto [31,32]. All animals used were kept in water at 20°C under a 12 hour light: dark cycle, and fed green leaf lettuce twice a week. Two-month old animals having a shell length of ~20 mm were used for the experiment.

### Surgical procedures and in vivo nerve injury

Snails were anesthetized by injecting 0.5 - 1 ml of 60 mM MgCl_2 _into the foot. In sterile snail saline containing (in mM): NaCl 51.3; KCl 1.7; CaCl_2 _4.1; MgCl_2 _1.5 (pH was adjusted to 7.9 with 1 mM HEPES/NaOH) a small incision was made in the dorsal head region and pinned open, exposing the cerebral and buccal ganglia. In the crush-operated animals, all right parietal and cerebral nerves were crushed using forceps [12]. Sham-operated animals were operated in the same way as the crush-operated groups, except that the right parietal and cerebral nerves were not crushed. Following the operation, both the sham-operated and crush-operated animals were maintained under standard laboratory conditions.

### RNA extraction and cDNA synthesis

Snails were anesthetized with 10% (v/v) Listerine for 10 min, following which the CNS including the buccal ganglia were dissected out for total RNA extraction at 1 hr, 3 hrs or 5 hrs post-injury. For the microarray there were 4 groups of sham-operated and 4 groups of crush-operated animals each with 2 snails per group. For RT-PCR 5 new groups of crush-operated and 5 new groups of sham-operated snails were used with 2 snails per group. We used TRIzol reagent and a modified protocol (Invitrogen, USA). 100 μl of Trizol was used per two excised CNS and buccal ganglia, and the final pellet was resuspended in 10 μl of RNase free water. RNA quality was checked on an agarose gel prior to cDNA synthesis and RNA concentration was measured using spectrophotometry. First strand synthesis of cDNA was conducted using SuperScript III reverse transcriptase (Invitrogen, USA) with random hexamer primer (Fermentas, USA) in a reaction volume of 20 μl for 1 μg of total RNA.

### cDNA Microarray

A microarray chip was designed based on the *L. stagnalis *CNS EST database http://www.Lymnaea.org/. The 15 K gene chip (cat#: G2509F) was made by Agilent Technologies (U.S.A). Briefly, the array contained a total of 15,000 spots, 10,333 probes were from unique EST/nucleotides and 4,425 probes included different sequences from the duplicate genes, and 50 technical replicates with 9 replicates per probe. The hybridization was performed by the University Health Network Microarray Centre (Toronto, Canada). One-Color Microarray-based Gene Expression Analysis (Agilent technologies) with cyanine 3-labeled targets was used to measure gene expression in experimental and control samples. Briefly, 500 μg of total RNA were amplified using a Fluorescent Linear Amplification Kit (Agilent Technologies) and labeled with Cy3-CTP and hybridized to the *L. stagnalis *cDNA microarray at 65°C for 17 hours. The images were scanned using a Genepix 4000B microarray scanner (Axon Instruments, Foster City, CA, USA). Image analysis, spot quality control and normalization were performed using the Feature Extraction software 9.5.3 from Agilent Technologies. The intensity files were loaded into GeneSpring GX 10.0.2 software (Agilent Technologies), a 75th percentile signal value was used to normalize Agilent one-color microarray signals for comparisons between arrays. The signals were log 2 transformed, and the median of the replicated probes was obtained. An unpaired t-test was performed and p < 0.05 was used as a cutoff; from these results we used a 2.0-fold change in signal intensity as a cutoff line to consider the differential expression of a gene as significant.

### Real-time quantitative PCR (qPCR)

Real-time PCR was performed using 5 μl of SYBR Greener Reagent System (Invitrogen), added to 1 μl of the appropriate primer set, and a diluted sample of cDNA that was topped off with DEPC water to a final volume of 10 μl. For the qPCR validation and time-course a new set of RNA samples from sham and crush-operated snails was used. Glyceraldehyde-3-phosphate dehydrogenase (GAPDH) has been used as a common control gene for spinal cord injury models [40,67], and its expression was unchanged following CNS injury in *L. stagnalis*, therefore, we used it as an internal control (Figure 3B). The cycling parameters were: 95°C/5 min, 40 cycles of 95°C/0.5 min, 55°C/1 min, followed by a melting curve protocol. The primer sequences used in the study are as follows: GAPDH 5'CTGCTGATGCCCCTATGTTTG/5'GTTGGTGGTGCAAGACTGCATT; C/EBP 5'CCTCTCATACATCTCCAAGTGC/5'GAGATGTTAGGGTGTAGGAATGG.

Changes in gene expression levels following CNS injury were determined using Ct-Ct plots [30], this analysis is a modification of the ΔΔCT and efficiency correction methods [68]. As described in our recent report [30], the efficiency of the amplification is assumed to be independent of the sample, and the standard curves from all the samples are linear. Assuming that the PCR efficiencies are independent of the sample, a Ct-Ct plot is created by plotting the Ct (threshold cycle) values of the test gene against the control gene. This yields a linear correlation between the gene pair and the slope (m) of the plot is described as m = log(E_c_)/log(E_g_). The Y axis of these plots is described by Ct_g _= m × Ct_c _- log(R)/log(E_g_); where R is the relative expression level between the target gene (Q_g_) and control gene (Q_c_); E_g _is the amplification efficiency of the target gene; Ct_g _and Ct_c _are the threshold cycles of the target gene and control gene, respectively [30]. The Y-intercept, a quotient of - log(R)/log(E_g_), is as a measure of R, because E_g _is constant. Thus, a small change in the Y_int _in the injury group over the control group indicates a decrease in the relative target-gene expression level.

### siRNA construct

Based on *L. stagnalis *C/EBP sequence, we designed a 27-*mer *siRNA using SciTools RNAi Design online software (IDT DNA). When choosing siRNA we selected for sequences with moderate to low G/C content, biased towards the 3'-terminus and purposely avoided sequences encoding the transmembrane domains [69]. The selected sequences were then purchased from Shanghai GenePharma Co. Ltd. The sequences for each siRNA are as follows: C/EBP siRNA#1: 5'CUAACAUCUCCUACUCAU UCCCAAA/5'UUUGGGAAUGAGUAGGAGAUGUUAGGG; C/EBP siRNA#2: 5'GGAUUAUAGUCAACAAAC/5'CUGCCUAGUUUGUUGACU; Control siRNA: 5'UUCUCCGAACGUGUCACGUTT/5'ACGUGACACGUUCGGAGAATT. For whole animal knockdown experiments snails were anaesthetized with 10% (v/v) Listerine, following which 2 μl of 20 μM control siRNA or C/EBP siRNA was injected into the head, above the central ganglia using a microlitre syringe (Hamilton Company, Reno, NV, USA) [70]. To confirm the siRNA knockdown using RT-PCR the ganglia were removed 48 hrs post-injection for RNA extraction (Figure 4C).

### Quantification of locomotion activity

Sham or crush operations were performed 48 hrs after injection with either saline, control siRNA, or C/EBP siRNA into the central ganglia region. Individual *snails *were placed in a large Petri dish (10 cm diameter, 2 cm depth) in 15 ml of water from their home aquaria. The snails were given 5 minutes to acclimate to their environment, and then were recorded for 10 minutes using Virtual Dub 1.9 software. The distance that each snail has crawled in 10 min was analyzed using ImageTool 3.1 software (UTHSCSA, Texas). Locomotion was analyzed prior to the surgery, and at day 1, day 5 and day 10 post operation.

### Primary cell culture

Snails were anaesthetized for 10 minutes in 10% (v/v) Listerine, and the central ring and the buccal ganglia were excised in snail saline containing (mM): NaCl 51.3, KCl 1.7, CaCl_2 _4.1, MgCl2 1.5, HEPES 2 (adjusted to pH 7.9 using 1 mM NaOH). The ganglia was then incubated in 3 mg/ml trypsin (Type III, Sigma, Ontario, Canada) for 24 min. PeA neurons, which are involved in locomotion [71], were identified and isolated from pedal ganglia with gentle suction using a Sigmacote (Sigma, Ontario, Canada)-coated fire-polished pipette (2 mm, WPI, 1B200F) as previously described [72,73]. The neurons were then placed onto a poly-L-lysine coated culture dish and maintained in diluted conditioned medium (CM) at room temperature [72-74].

### Axotomy and neurite outgrowth

PeA cells were cultured in CM at room temperature for 24 hours, allowing for sufficient neurite outgrowth. Transection of the neurite was then performed using a glass micropipette, controlled by a micromanipulator, and moving it perpendicularly across the neurite until a complete cut was observed [47,75]. Immediately following axotomy the cells were treated with either the brain conditioned medium (CM) or a final concentration of 7 nM C/EBP siRNA#1, C/EBP siRNA#1 siRNA or control siRNA in a final volume of 2 ml of the CM. Images of neurites were collected at the time of RNAi treatment (t = 0), and over a 36 hr period at three time points (10, 24 or 36 hrs), using an inverted microscope (40× objective) (Olympus CK X41) with a digital camera (Olympus C5050). The lengths of the proximal neurite, the distal neurite and an intact neurite of the axotomized cell were measured at various time points, using ImageTool 3.1 software (UTHSCSA, Texas).

### Statistics

Data are presented as mean ± S.E.M. Data were imported to Microsoft Excel or OriginPro v8 (Silverdale Scientific Ltd., Bucks, HP, USA) for graphical presentation. Statistical significance between mean values of experimental groups was evaluated using a Student's t-test for two groups and ANOVA with the Holm-Sidak post hoc test for multiple comparisons, using SigmaStat 3.0 (SPSS, Chicao, IL, USA). Significance was defined by probability level of lower than p < 0.05.

## Authors' contributions

ZPF conceived, designed and directed the study. MA conducted the experiments and dada analysis. ZPF and MA wrote the manuscript. ZPF and MA read and approved the final manuscript.

## Supplementary Material

Additional file 1 **The raw microarray data from 4 independent sets of experiments**. The original data obtained from microarray analysis of the ganglionic mRNAs obtained from 4 sham-operated and 4 crush-operated snails.. The highlighted line indicates C/EBP mRNA expression levels. Red: indicates the genes with 2 fold changes in expression level in crush-operated over sham-operated snails. Click here for file
